# Perceived Barriers to Success for Minority Nursing Students: An Integrative Review

**DOI:** 10.5402/2012/806543

**Published:** 2012-05-30

**Authors:** Collette Loftin, Susan D. Newman, Bonnie P. Dumas, Gail Gilden, Mary Lou Bond

**Affiliations:** ^1^Department of Nursing, West Texas A&M University, Canyon, TX 79016, USA; ^2^College of Nursing, Medical University of South Carolina, Charleston, SC 29425, USA; ^3^College of Nursing, University of Texas, Arlington, TX 76019, USA

## Abstract

The objective of this paper was to identify barriers to successful program completion faced by underrepresented minority nursing students. This paper reveals that minority nursing student's face multiple barriers to success including lack of financial support, inadequate emotional and moral support, as well as insufficient academic advising, program mentoring, technical support, and professional socialization. An additional theme—a resolve to succeed in spite of the identified barriers—was identified. This body of literature focuses solely on successful minority students' experiences, revealing a significant gap in the research. The findings of this paper highlight the need to create and maintain nursing programs capable of aggressively supporting minority student needs. Recommendations for future research are included.

## 1. Introduction

The increasing diversity of the United States is evidenced by a recent census report predicting that by midcentury, racial and ethnic minorities will make up over half of the United States' population [1]. A culturally diverse nursing workforce is critically needed to meet the healthcare needs of this increasingly diverse population. Recruiting and retaining underrepresented minority (URM) nursing students continues to be an important component of this process but remains a challenge for the nursing education community. National nursing and healthcare organizations including the National League for Nursing (NLN), the American Association of Colleges of Nursing (AACN), the Institute of Medicine (IOM), and the American Nurses Association (ANA) agree that increasing the racial and ethnic diversity of students in US nursing programs is a high priority [2–5]. By increasing recruitment and improving retention and graduation rates of minority nursing students, nursing programs could facilitate increased minority representation in the nursing workforce.

## 2. Background and Significance

Registered nurses represent the largest number of professional healthcare workers in the United States. However, the racial and ethnic background of the nursing workforce is not reflective of the general population as a whole. Like other healthcare professions, minority representation in nursing is lagging [4]. Initial findings from the 2008 Sample Survey of Registered Nurses report that although the minority population constitutes 37 percent of the country's population, minority nurses make up only 16.8 percent of the total nurse population [6]. In addition to being the largest group of healthcare providers, nurses work in virtually all healthcare and community settings. Consequently, changes in the ethnic and racial composition of the nursing workforce have the potential to influence healthcare across all areas and settings.

Perhaps the most compelling reason for increasing the numbers of underrepresented minority nurses is the lack of equity in regard to healthcare access and quality. The groundbreaking Institute of Medicine (IOM) report *Unequal Treatment* documented disparities found in the access to and quality of the healthcare provided to racial and ethnic minorities [7]. Race and ethnicity play significant roles in the care minority patients receive even when other factors are considered equal such as health insurance coverage and income [7]. Studies show that minority Americans are much more likely to die in infancy, suffer higher rates of chronic diseases, and have shorter life spans than majority Americans [8]. According to the Sullivan Commission [9], “The rationale for increasing diversity in the health workforce is evident: increased diversity will improve the overall health of the nation” (page 3).

Efforts to increase diversity in nursing programs have resulted in increased admission of minority students. According to the AACN [10], the percentage of students from URM backgrounds in prelicensure baccalaureate programs during 2010 was at its highest rate ever—26.6%. Historically however, high attrition rates have been a significant concern [11–13]. There is a dearth of research exploring the association between attrition and diversity [14, 15]. While there remains a lack of consistent documentation regarding attrition rates, estimates for minority nursing students have ranged from 15–85 percent [11, 13, 16].

The Health Resources and Services Administration (HRSA) Nursing Workforce Diversity program has awarded millions in funding for projects to increase nursing education opportunities for students from racial and ethnic minorities underrepresented among registered nurses [17]. The funding provides student stipends, enrichment and recruitment programs, and retention activities. In 2009, 51 workforce diversity grants were funded providing $16 million in aid [5].

## 3. Purpose

There has been continued demand for increased diversity in nursing thus a review of current literature regarding the barriers to successful program completion is warranted. Improved awareness about the barriers will enable educators to direct future efforts toward interventions that effectively impact and facilitate successful program completion for underrepresented minority nursing students. The purpose of this paper is to integrate the findings from recent studies identifying perceived barriers to nursing program completion for URM nursing students.

## 4. Methodology

 This integrative review follows the five stages of review as proposed by Whittemore and Knafl [18]. These stages include problem identification, literature search, data evaluation, data analysis, and presentation. There has been much published in nursing journals offering expert opinion and suggestions to increase diversity in nursing education and in the nursing workforce. This paper is limited, however, to the published research reporting barriers to successful program completion for URM students in prelicensure nursing programs.

A comprehensive search of the literature was performed to locate such articles published between 1996 and 2011. This search was completed in early spring of 2011. The following online databases were utilized in this search: Cumulative Index of Nursing and Allied Health Literature (CINAHL), Educational Resources Information Center (ERIC), EBSCO, and Pub Med. The following search terms were used alone and in combination: *nursing workforce, diversity, nursing student, ethnicity, minority, nursing education, barriers, and underrepresented minority*. Once a study was identified, the author(s) were added as additional search terms. Additional articles were identified through a hand search of reference lists. This search strategy yielded 613 articles.

Titles and abstracts were reviewed in depth to determine studies' relevance for inclusion in this paper based on the following criteria. Exclusion criteria included studies published prior to 1996, studies conducted outside the U.S., or articles that were not published in English. Studies that were limited to minority student experiences in graduate nursing programs were not included. Commentaries, expert opinion pieces, and review articles were also excluded. Seventeen publications met the selection criteria and were included in this review. Once identified, results of the studies were carefully reviewed and analyzed. Analysis was aided by presentation in a table format under the headings of author/date, study purpose, study design, sample, and findings.

## 5. Conceptual Framework

The Adapted Model of Institutional Support conceptually informed this review. The Model of Institutional Support [19] was originally developed to describe institutional supports necessary to assist Hispanic doctoral students. Valverde and Rodriquez based this model on a review of the literature and a retrospective examination of their own experiences as doctoral students. The constructs of their original model included financial support and opportunity, moral and emotional support, faculty and professional mentorship, and technical support.

Valverde and Rodriguez introduced the significance of academic advising in their initial mentorship construct. Bond et al. adapted the model by extracting academic advising into a fifth construct [20]. They further revised the model to include a sixth construct. Professional socialization was originally identified by Conway as vital for graduate students [21]. The construct was considered similarly important for undergraduates by Bond and colleagues and subsequently added as the sixth construct.

## 6. Results

A majority of the reviewed studies (16 of 17) utilized a qualitative methodology employing, open-ended interview, focus groups, or questionnaires that included open-ended items to explore and describe the students' experiences in a prelicensure nursing program. Both associate degree and baccalaureate degree student perspectives were included in the reviewed studies. Similarity was noted in the studies' stated purpose which often sought to identify personal and/or educational barriers encountered by the students as well as explication of their experiences in predominately White schools of nursing. The reviewed studies are summarized in Table 1. The barriers identified in this paper are organized by the concepts of the Adapted Model of Institutional Support.

### 6.1. Financial Support

One of the major barriers identified by students was financial with many reporting that it was necessary to work in order to make ends meet [11, 22–28]. For some, the need to work extended their time in the nursing program [29]. Interestingly, students in the Loftus and Duty study considered the need to work as few as 1–12 hours per week to be a significant barrier to their academic success [28]. Working long hours decreased the amount of time available to study for students [11, 22, 23, 26].

Two common reasons minority students gave for working included the need to cover education expenses as well as to provide for the living expenses of themselves and their families [20, 23, 25, 26, 30]. The rising cost of tuition was considered an additional burden and added to minority students concerns. In an effort to decrease the financial concerns of rising tuition, some of these students chose to enroll in community colleges for prerequisite courses instead of universities. Others identified the lack of financial aid and pressure to begin earning money as reasons for enrolling in community college nursing programs instead of university programs [26, 29, 30].

Student's financial challenges were further complicated by the fact that they had been provided with little or no financial aid information regarding scholarships and grants [25, 26]. Most considered loans to be the only available avenue for financing their education. Moreover, these students reported that their instructors had recommended they work fewer hours or stop working altogether [25, 26].

### 6.2. Emotional and Moral Support

A lack of emotional and moral support was perceived to be a key barrier by minority students in most of the studies. This lack of support manifested itself in multiple ways including feelings of social isolation and loneliness, racism and discrimination, and, finally, through family support issues.

#### 6.2.1. Isolation and Loneliness

Insufficient numbers of African American students in nursing programs decreased the opportunities for students to connect and develop relationships with other African American students leading to feelings of social isolation and loneliness [12]. The same was found for Hispanic students [26, 29], Native American students [31], and international students [23]. While initially identifying a lack of other minority students in their programs as “very hard,” some were eventually able to overcome these feelings as their program grew in size and more minority students were admitted [27].

Hispanic, Native American, African American, Asian, Nigerian, and Eastern Indian students described feeling isolated and lonely both inside and outside the classroom [12, 16, 23–27, 29, 31–33]. These students described being the last person chosen as a lab partner and being unable to find classmates willing to form study groups with them [32].

#### 6.2.2. Discrimination

Multiple studies provided accounts of students being subjected to discrimination and racism by faculty, preceptors, peers, hospital staff, and patients [12, 16, 22, 24, 26, 27, 29, 31, 33, 34]. In one example, a Latina student described being told by a male patient that he wanted a White nurse to care for him [22]. Another recounted a patient assuming she must be the nursing assistant and not the nursing student while presuming the blond nursing assistant to be the student nurse. Another student described a patient refusing to allow a Hispanic nursing student to care for her saying, “I do not want her to touch me” [27].

The perception that nursing faculty members have a negative bias toward minority students was expressed in many of the studies. One student reported, “I was actually confronted by one of my instructors…she took me to her office with no one listening [she told me] that she did not like my face, and she was going to make it extremely difficult for me at school. And she did” [29]. The perceived discrimination encountered by Hispanic participants in the same study was described as “horrific, painful, and frequent.”

African American students described their environment as “adversarial and nonsupportive” explaining that they were required to “excel 10 times more and be more presentable [dress] even on my worst days” [12]. Another student recounted that she did not believe the White faculty “even knew our names” (page 10). Similarly, others described being misunderstood by classmates and faculty, intimidated, and negatively viewed as “lazy” or “dumb” [11, 31].

Students described a “weeding out” process in which faculty members targeted minority students for failure [30]. This process was characterized as identifying a student's academic and clinical weakness and then targeting questions toward that weakness. One student's description was, “A student who had difficulty responding quickly to questions about medications they were to give would be grilled even harder. Each time that student would be in a clinical; they would be singled out for an intense questioning about their assignment for the day. This would happen to several students and by the end of the semester they would be gone” [30].

#### 6.2.3. Family Issues

 Issues regarding family support were identified as both facilitators and barriers [26]. For some students, family members were described as less supportive because of gender role stereotyping [11]. Families of Hispanic female students did not consider it as important for them to complete their education as they did for their male family members [20].

Female students also struggled with conflicting expectations and the responsibilities of caring for their families and extended families and their studies [16, 24, 26, 29]. Meeting family obligations and caring for their children and homes was found to be of great importance even to the extent of sacrificing study time [16, 20, 22, 24, 25, 27]. Students who missed family functions to study or attend class were subjected to criticism and negative feedback from family members [22]. Role stereotyping was identified as a barrier for Hispanic males too. Nursing was often considered a female vocation and not necessarily an appropriate career choice for Hispanic males [20, 26].

### 6.3. Advising and Academic Support

Minority students perceived the nursing program to be “much more difficult” than they had expected in sharp contrast to the Anglo students in one study [27]. Similarly, others reported having no comprehension of what would be required of them academically to succeed in the nursing program [26]. The stereotypical image of nursing had led them to believe they would learn how to give medications to patients and a few other procedures. Instead, these students were overwhelmed after realizing this was only a small portion of what they were required to learn.

Students in the Amaro et al. [22] study believed that they had not received adequate support to learn required academic material and reported that they were unaware of tutoring or other support services available to them as nursing students. Students in the reviewed studies reported that the nursing program workload and pace interfered with their academic performance more frequently than nonminority students [26, 28]. The lack of flexibility in scheduling classes and clinical courses was considered an additional barrier for some students [29].

A lack of knowledge regarding the requirements for admission to nursing programs was identified as an added barrier [20, 24, 26, 32]. The lack of appropriate advising during high school and early college years about GPA and prerequisite course requirements was also acknowledged as problematic. For some, the lack of preparation was due to the schools' focus on students meeting requirements for graduation rather than preparation for success in college [24].

### 6.4. Mentoring

Lack of minority faculty to serve as mentors and role models was identified as challenging for diverse students. Students reported believing that a mentor would help them to be more successful in their nursing program [33]. They identified minority faculty members as easier to approach. Students described feeling uncomfortable approaching a nonminority faculty member as well as perceiving a hesitancy on the part of nonminority faculty members to engage in relationships with them [33]. In addition, these students sought out relationships with minority nurses they encountered in their clinical experiences. These sentiments were echoed in other studies [22, 25].

### 6.5. Professional Socialization

Ethnic minority student associations were reported to be very helpful to student members by providing motivational support and information about classes and strategies to improve academic performance [22]. In addition, membership was found to provide social and professional support. Unfortunately, such groups are often unavailable [22]. When available, many students were not aware of their existence [34]. Regrettably, some students reported having too little time for involvement in student organizations [20].

### 6.6. Technical Support

Computer access and technology competence were reported in only a single study as significant barriers to successful academic performance [28]. Students reported struggles with accessing a computer for use at home as well as having access to an internet connection to complete assignments and informational searches. Minority students in this study also identified the inability to type and not being knowledgeable or skillful at using basic software applications such as Microsoft Word as additional barriers.

### 6.7. Other Themes

#### 6.7.1. Resolve to Succeed

Eight of the reviewed studies described an additional theme—a resolve to succeed in spite of the barriers. While not a concept of the Adapted Model of Institutional Support, Bond and colleagues discussed integrating such a concept in their 2008 study [20].  Table 2 provides a description of the determination/perseverance theme from each of the eight studies.

#### 6.7.2. Cultural Competence

Minority students in several of the reviewed studies discussed their White peers' lack of cultural knowledge and competence as a barrier for them [16, 31, 34]. Students expressed feeling exasperated by their classmates' limited cultural knowledge, which they attributed to a lack of exposure to diversity [24]. Others attributed it to being “uneducated” or “uncomprehending” [34]. Some students indicated limited cultural content in the program's curricula as a concern [31].

## 7. Discussion

 While most of these reviewed studies were small, together they begin to paint a picture of the barriers to success many minority nursing students experience during their undergraduate nursing education. These are in addition to the typical challenges majority nursing students encounter. This integrative review provides an overview of the nursing research aimed at identification of those perceived barriers. Results of the review indicate that the successful completion of a nursing education is complicated and there are numerous barriers to overcome for minority students.

Financial struggles were common to the student participants in the reviewed studies. Unfortunately, for nursing students who consider financial concerns to be among their most difficult barriers, there is not likely to be a great deal of new aid available. As a result of the recent economic downturn and significant deficiencies in most state budgets, state support for higher education has been decreasing. Identification and dissemination of information on available state and federal financial aid through the nursing program will benefit all students.

Mentoring programs and opportunities for professional socialization can become facilitators of success and achievement for diverse students. The transition from student to nursing professional requires planning and active participation both inside and outside the classroom. Minority students in this review expressed a hesitancy to approach nonminority faculty, suggesting that they could more easily approach minority faculty, but often there are too few minority faculty members to serve as mentor and advisor. The development of formal mentoring and professional socialization programs will provide safe avenues that students can utilize to reach out to all available faculty mentors and advisors.

Support for technical and computing competence concerns was mentioned in only two of the studies. Computer and technical competence are fundamental skills for nursing students and essential for successful program completion. In a study assessing nursing students' computer skills, Elder and Koehn [35] found that while students possessed some of the skills necessary in a computerized environment, they lacked many of the skills required to complete course work including word processing skills. These students had assessed their technical skills to be very high while their actual assessment score showed their skills to be marginal. Elder and Koehn have recommended that student computer competency be assessed early, permitting accurate course advising about the need to complete a computer course prior to beginning upper level nursing courses.

The findings from this review have implications for nursing faculty and program administrators. Commitment to the retention and program success of diverse nursing students from faculty and administrators can significantly reduce some of the barriers they face. Data from the reviewed studies suggests that nursing faculty can have a negative effect on minority students' educational experience through prejudicial and discriminatory practices. Therefore, faculty's appreciation of the influential role they have in educating diverse nursing students is essential.

A substantial gap, noted in this reviewed body of literature, is that no study included participants who had been unsuccessful or left their nursing program prior to graduation. The literature focused solely on barriers faced by successful students. In fact, several of the studies included nurses who had already graduated and successfully completed the NCLEX-RN exam. Minority and ethnic nursing students who are not able to persist through to program completion may have dissimilar needs or additional barriers to success that were not identified by those students successfully completing nursing programs. Locating these students for participation in future studies will likely be difficult, but every effort should be made to include the perspective of this population.

Further study of the *Resolve to Succeed/Determination to Succeed *theme is warranted. Identification of methods to enhance the determination and resolve of minority students to succeed would further aid program directors and faculty members to design effective strategies to promote success.

## 8. Conclusions

The number of published articles discussing the need to increase diversity in the nursing workforce provides evidence that this topic is not being ignored. Unfortunately, the undertaking has met with minimal success as yet and in some aspects nursing education has not been responsive to the dilemma of URM nursing students as evidenced by the slow growth in numbers of URM nurses. Minority nursing students face daunting barriers and there is much to be done to improve the educational process for them. The findings of this paper highlight the need to create and maintain nursing programs capable of actively and aggressively supporting minority student needs as well as facilitating a climate that is welcoming and caring.

## Figures and Tables

**Table 1:** Presentation of reviewed studies.

Author and date	Study purpose	Study design	Sample	Findings
Jordan (1996) [34]	Examine lived experiences of AA students in mostly White BSN program	Hermeneutics study—interviews	4 AA nursing students	Three patterns emerged—*Seeking Identity:* being only AA student in the class led to feelings of representing entire race; *Student as Teacher*: nursing curricula did not always include cultural differences and was not inclusive; *Resoluteness: *forced to persevere even in the face of racism, ignorance, and myths

Villarruel et al. (2001) [29]	Identify barriers and bridges to educational mobility	Qualitative methodology utilizing focus groups	37 Hispanic nurses participated in focus groups	Financial considerations required participants to work while pursuing education and reason many choose ADN program. Institutional barriers included unsupportive faculty, perceived discrimination by faculty and peers, lack of advisement, and lack of scheduling flexibility. Cultural/family barriers included family care giver or wage earner responsibilities

Weaver (2001) [31]	To describe supports and struggles endured as student	Survey	40 Native American nurses and nursing students	Barriers identified included culture shock or differences, endured stereotypes and racist attitudes, isolation, and assumption about cultural identity

Sanner et al. (2002) [23]	Explore the experiences of international students in BSN program.	Guided interview approach	8 Nigerian nursing students	All students reported becoming socially isolated and being prewarned of the social and academic challenges of the nursing program. Further identified the need to work to pay academic and living expenses

Perez (2003) [30]	To build understanding of the nursing education choices made by participants	Quant./qual. utilizing questionnaire and interviews	Qualitative—20 Mexican Americans—all female Quantitative—485 responses years	Numerous barriers were identified including financial difficulties and need to be employment, nursing education considered more difficult than expected, stress on family priorities and commitments

France et al. (2004) [32]	Explore the lived experiences of AA students in a mostly White rural university	Phenomenological approach—interviews	4 AA nursing students	Three themes emerged including—*You're just shoved in the corner*: lack of relationships with other students, lack of collegiality and lack of support among classmates*, I have to strive to do the best I can:* students set high expectations for themselves, and finally*, “*…* You just got to maintain”*: students expressed persevering in spite of obstacles

Gardner (2005a) [16]	Identify the factors influencing success of foreign-born nursing students	Case study	East Indian nursing student	Identified barriers included family demands and obligations, feeling of exclusion and isolation with regard to peers, and cultural differences including a reluctance to speak up in class or make eye contact with faculty

Gardner (2005b) [24]	To document minority nursing students' perspectives of their experiences in a mostly White program	Phenomenological framework utilizing semistructured interviews	15 racially and ethnically diverse students	Emerging themes included—*Loneliness and Isolation* from majority students; *Differentness*: feeling “different” from majority students; *Absence of Acknowledgment* from faculty; *Lack of Understanding* from peers about cultural differences; *Desired Support from Faculty*—but often did not receive it; *Coping with Insensitivity and Discrimination; Determination to build Better Future *

Mills-Wisneski (2005) [33]	To examine AA BSN students' perception of impact of absence of minority faculty	Descriptive design-questionnaire utilizing Likert-type scale and open-ended questioning	71 AA students from 9 programs	Students expressed concerns about lack of minority role models and difficulty establishing relationship with majority faculty as well as perception of being treated differently by White faculty

Amaro et al. (2006) [22]	To determine diverse nursing students perceptions of educational barriers	Grounded theory methodology utilizing in-depth interviews	17 ethnically diverse recent graduate RN's	Student needs and barriers included: lack of time due to family responsibilities, lack of adequate finances, major academic difficulties—some related to academic background and language, and prejudice and discrimination

Taxis (2006) [11]	To explore the experiences, perceptions, and factors that influence retention and graduation from a mostly White BSN program	Qualitative methodology utilizing questionnaire and face to face interviews, and focus group	Nine Mexican-American students in a predominately White BSN program or recent graduates	Identified barriers included financial situations that required working which resulted in inadequate time for rest, exercise, and study, not fitting in with Anglo peers, and difficulty functioning in two separate cultures including their predominately White university and their Mexican American peers and family

Goetz (2007) [26]	Identify the barriers faced by students completing studies and to identify strategies used to manage or overcome barriers	Qualitative methodology utilizing interview—grounded theory approach	12 Hispanic nurses and nursing students	Numerous barriers identified including: being ill-prepared for the difficulty and intensity of the nursing program, time management problems balancing home, school and work responsibilities, financial issues, family beliefs and cultural influences, inadequate academic preparation, and prejudices

Rivera-Goba and Nieto (2007) [25]	To explore meaning and significance of mentoring during prelicensure program for Latinos in nursing	Phenomenological framework utilizing semistructured interviews	17 Latina students or recent graduates	Marginalization was identified as a major challenge; feeling isolated or as if they lacked knowledge. Financial burdens were identified as a barrier. Faculty suggested students decrease amount of work although students needed to pay educational expenses as well as family expenses

Bond et al. (2008) [20]	To identify perceived barriers and support for retention in nursing program	Qualitative—focus group studies with theory guided data analysis	14 Mexican-American students All Hispanic	Findings included revealed barriers congruent with model components of financial issues, emotional and moral support issues, professional socialization issues, mentoring, technical support, and academic advising issues

Coleman (2008) [12]	To explore academic and social experiences of AA students enrolled in a mostly White institution	Qualitative methodology utilizing semistructured interviews	14 African-American students from a two-year community college	Four primary themes emerged including difference, coping, and survival, support systems, and institutional context as having significant influence on the experiences of the students

Evans (2008) [27]	Compare and contrast perceptions concerning barriers between minority students and a comparison group of Anglo students	Descriptive qualitative utilizing semistructured interviews	14 Hispanic and American Indian students	Barriers to success included: financial hardships and work-related issues, heavy family obligations, early difficulty relating to nonminority students and faculty, fear of academic failure, and lack of faculty contact

Loftus and Duty (2010) [28]	To determine facilitators and barriers to successful completion of a BSN program	Naturalistic utilizing survey	314 student participants: White—276 Black/AA—16 Other minority—17	Five barrier factors were identified including English language and computer skills, workload and pace of program, financial and family concerns, technology access (owning a computer or having internet access), and technology competence

Table 1 identifies author(s), study purpose, sample, and brief description of findings for each study.

**Table 2:** Resolve to succeed theme.

Author	Theme	Description
Jordan (1996) [34]	Resoluteness	Resilience in the face of challenge; remaining steadfast to educational goal despite overwhelming trials
Sanner et al. (2002) [23]	Persistence despite perceived obstacles	Placed the burden on selves to make the necessary adjustments as they attempted to assimilate
Frances et al. (2004) [32]	“…you just got to maintain”	The participants persevered in spite of feeling isolated and discounted by others
Mills-Wisneski (2005) [33]	Persistence and self-motivation	Absence of minority faculty served as a catalyst and motivating factor to succeed in nursing program
Gardner (2005b) [24]	Determination overcome obstacles	Success is necessary to build a better future; Desire to overcome despite many obstacles
Amaro et al. (2006) [22]	Self-motivation and determination	Participants determined to complete their courses; strength was demonstrating self-motivation and determination
Rivera-Goba and Nieto (2007) [25]	Perseverance	In spite of many roadblocks, perseverance was the driving force to succeed; had an overall desire to succeed
Bond et al. (2008) [20]	Personal determination	Showed a personal determination to succeed and a desire to give back to other Hispanic students still “in the pipeline”

Table 2 provides a description of the *Resolve to Succeed/Determination Theme* identified in eight of the reviewed studies.
